# Learning Outcomes of Diverse Oncology Professionals After the TEAM Cultural Competency Training

**DOI:** 10.1007/s13187-020-01865-4

**Published:** 2020-09-09

**Authors:** Mandi L. Pratt-Chapman

**Affiliations:** 1 The George Washington University School of Medicine and Health Sciences, Washington, DC USA; 2 The GW Cancer Center, The George Washington University, 2600 Virginia Ave, #324, Washington, DC 20037 USA

**Keywords:** Cultural competency training, LGBTQI, Racial and ethnic minorities, Health care professional education, Evaluation, Communication

## Abstract

Racial, ethnic, sexual, and gender minorities are more likely to report challenges with oncology provider communication and quality of care. The Together-Equitable-Accessible-Meaningful (TEAM) training was developed to improve health equity across cancer care organizations by guiding teams of interprofessional learners through planning and implementation of quality improvements to advance equitable, accessible, and patient-centered cancer care. This study compared changes to self-reported cultural competence as measured by the Cultural Competency Assessment (CCA); Lesbian, Gay, Bisexual, and Transgender Development of Clinical Skills Scale (LGBT-DOCSS); and Interprofessional Socialization and Valuing Scale (ISVS). The primary aim of the study was to assess changes to self-reported cultural competence; the secondary aim was to examine changes to interprofessional valuation from baseline to post-intervention. Results indicated statistically significant improvements in self-reported Cultural Competency Behaviors (*p* = .055), a subscale of the CCA, and Attitudinal Awareness toward sexual and gender minorities (*p* = .046), a subscale of the LGBT-DOCSS, using *p* < .10 as statistically significant. These subscale results drove statistically significant improvements for their respective composite scales. No other statistically significant results were found. This study suggests that cultural competency training among interprofessional oncology health care professionals can be effective. Given the growing diversity within the USA, additional opportunities for cultural competency training are needed.

## Background

Racial, ethnic, sexual, and gender minorities in the USA are likely to encounter few oncology health care professionals who share their lived experiences: while Black and Latino Americans comprise 13% and 18% of the US population, respectively [1], the oncology workforce is disproportionately White (52%) with few Black (3%) or Hispanic (5%) physicians [1, 2]. Minorities are more likely to report poor provider communication, lower quality of care, and lower self-efficacy [3–5]. Sexual and gender minorities experience greater risk factors for some cancers and are more likely to report negative experiences with health care providers, fear of discrimination, low levels of trust in the provider, breach of confidentiality, invasive and inappropriate questioning, and unmet needs [6–9].

The purpose of this study was to assess the effectiveness of a novel, hybrid online and in-person cultural sensitivity training called Together-Equitable-Accessible-Meaningful (TEAM) on a diverse group of oncology professionals. While there have been many calls to action for cultural competency training [10], there have been few evaluations of the effectiveness of training among health care professionals at large [11] or among cancer care professionals specifically [12]. In fact, until recently, the only published intervention evaluating effectiveness of cultural competency training among cancer care professionals was focused on clinical trial recruitment of racial and ethnic minorities [12]. There is emerging literature on training oncology providers to improve the care of sexual and gender minorities [13], but there are no known studies that have evaluated the effectiveness of cultural competency training using an intersectional approach among oncology professionals. The learning intervention for this study was novel by training multidisciplinary teams of cancer healthcare professionals using an intersectional approach in order to build competence within organizations more broadly. In this study, we examined changes to self-reported cultural competence generally, competence with sexual and gender minorities specifically, and changes to participant valuation of interprofessional learning.

## Methods

### Intervention Description

The TEAM training was created to improve health equity by supporting organizational changes at the systems level. The training aimed to guide cancer care organizations in the implementation of quality improvements to advance equitable, accessible, and patient-centered cancer care through improved patient-provider communication, cultural sensitivity, shared decision-making, and attention to health literacy. Training components included a self-paced, 5-h online course; three virtual technical assistance sessions to help teams prepare for the in-person portion of the training; a 2.5-day in-person workshop with a focus on the development of an organizational action plan to improve a cancer-related service to be more culturally sensitive and equitable; and three virtual technical assistance sessions to help address action plan implementation challenges. All components of the training were at no cost to participants.

#### Participants and Procedures

The study (IRB# 180719) was determined to be exempt from IRB review under DHHS regulatory category 2. Teams were selected through a competitive application process. Selected organizations (*n* = 12) included teams from American Samoa (*n* = 2), California (*n* = 2), Florida, Hawaii, Louisiana, Maine, Massachusetts, Michigan, Minnesota, and Montana. Individual learners (*n* = 47) were diverse in professional role, race, and ethnicity. Learners were not required to participate in the study to access the learning intervention. Learners ranged from 27 to 75 years of age (M = 45.94, SD = 11.456).

Data were collected at baseline (March 2019) and post-intervention (December 2019), stored in the secure RedCap database, and exported to SPSS 26 for analysis.

#### Survey Measures

The pretest survey included ten demographic questions. Both pre- and posttest surveys included the 25-item Cultural Competency Assessment (CCA) [14], the 18-item Lesbian, Gay, Bisexual, and Transgender Development of Clinical Skills Scale (LGBT-DOCSS) [15], and the 24-item Interprofessional Socialization and Valuing Scale (ISVS) [16].

The CCA is a 2-factor scale that measures Cultural Competence Behaviors and Cultural Awareness and Sensitivity. The CCA has demonstrated strong consistency reliability (*α* = .92) [14]. Response options for the original CCA included a 4-point response ranging from always (5) to never (1) and a fifth “no opinion” category (3) on the far right. Dillman (2000) recommended this approach to ensure a truly neutral option for surveys [17]. Factor 1 (Cultural Competence Behaviors) includes 17 items and factor 2 (Cultural Awareness and Sensitivity) includes 8 items.

The LGBT-DOCSS is an 18-item, 3-factor scale that measures Clinical Preparedness (7 items), Attitudinal Awareness (7 items), and Basic Knowledge (4 items). The LGBT-DOCSS has internal consistency of *α* = .86. The original 7-point scale of the LGBT-DOCSS was modified to a 4-point scale with a fifth “no opinion” option for cognitive consistency with the CCA. Eight items (3, 4, 5, 7, 9, 12, 17, and 18) were reverse coded per instructions from the author of the scale [15].

The ISVS is a 24-item, 3-factor scale that evaluates the degree to which transformative learning takes place in the context of interprofessional teamwork. Strong internal consistency of ISVS has been demonstrated (*α* = .90) [12]. The three factors of the ISVS include Self-Perceived Ability to Work with Others (9 items), Value in Working with Others (9 items), and Comfort in Working with Others (6 items). The ISVS original 7-point scale with a not applicable response was modified to a 4-point scale with a “no opinion” response to support cognitive consistency of all scales.

#### Data Preparation and Coding

The 7-point LGBT-DOCSS and ISVS scales were modified to align with the 4-point plus “not applicable” option used by the CCA. Due to the requirement to assign a number to each answer option in RedCap, the “not applicable” option was listed on the far right with zero corresponding points assigned while the 4-point scale for all measures was as follows: always (4), often (3), at times (2), never (1), no opinion (0). Prior to analysis, data were imported into SPSS 26 and recoded as follows: always (4), often (3), no opinion (2), at times (1), never (0). This coding downshifted all original scales, so the range for the CCA was 0–100 inclusive of Cultural Competence Behaviors (0–68) and Cultural Awareness and Sensitivity (0–32). The possible range for the LGBT-DOCSS was 0–72 inclusive of Clinical Preparedness (0–28), Attitudinal Awareness (0–28), and Basic Knowledge (0–16). The range for the ISVS was 0–96. For all scales, higher scores reflect greater self-identification with the construct being measured.

#### Missing Data

Responses to all items were voluntary which resulted in a high degree of missing data. Of the 47 learners who participated in TEAM, over half (*n* = 28) of participants completed some portion of the evaluation, and 36% of learners completed both pretest and posttest surveys (*n* = 17). Chi-square and *t* tests were conducted on demographic variables to examine between-group differences of those who completed pre- and posttests versus those who only completed a portion of the evaluation. Those who did not complete both surveys were more likely to report a greater number of training hours on cultural competence (*p* = .090) and a greater number of minority patients seen in the 6 months prior to the intervention (*p* = .003). Data were determined to be not missing at random, so cases were excluded by analysis rather than listwise to preserve as much data as possible. In pairwise deletion, cases are dropped only if there is a missing value in the variable being analyzed. This preserves the greatest power for the sample, but may result in different sample sizes for different variables measured.

#### Statistical Analysis

Frequencies were used to describe the sample (Table 1). Statistical significance was predetermined at *p* < .10 given the small sample size. Paired *t* tests were conducted to examine pre- to posttest changes for all composite and subscales.

**Table 1:** Characteristics of study sample

Participant characteristic	*n*	Full sample	Non-paired comparison group	Paired pre- and posttests sample used for study
Professional role	33			
Administrator		7	4	3
CHW		1	0	1
Navigator		6	1	5
Nurse		1	1	0
Nurse practitioner		1	1	0
Physician		5	5	0
Social worker		1	0	1
Other		11	5	6
Age	33	M = 45.94	M = 48.82	M = 42.88
		(SD = 11.456)	(SD = 10.388)	(SD = 12.060)
Sex assigned at birth	28			
Female		23	10	13
Male		5	2	3
Gender identity	28			
Female		23	10	13
Male		5	2	3
Non-binary gender		0	0	0
Other gender identity		0	0	0
Sexual orientation	28			
Heterosexual		26	12	14
Bisexual		0	0	0
Lesbian or gay		2	0	2
Other sexual orientation		0	0	0
Race/ethnicity*	28			
American Indian		1	0	1
Asian		0	0	0
Black		3	1	2
Hispanic/Latino		3	0	3
White		17	9	8
Other		5	2	3
Political affiliation	28			
Very liberal		6	2	4
Somewhat liberal		7	4	3
Neither liberal nor		9	3	6
conservative		5	2	3
Very conservative		1	1	0
Apolitical		0	0	0
Religious affiliation	28			
Agnostic		2	1	1
Christian (Catholic)		8	3	5
Christian (Protestant)		12	5	7
Jewish		1	1	0
Other		3	1	2
Prefer not to answer		2	1	1
Spirituality	28			
Not at all spiritual		0	0	0
Somewhat spiritual		7	3	4
Spiritual		13	6	7
Very spiritual		8	3	5
Religiosity	28			
Not at all religious		3	2	1
Somewhat religious		14	6	8
Religious		7	2	5
Very religious		4	2	2

*Not mutually exclusive

## Results

Participant characteristics are provided in Table 1. Each team was asked to include a clinician, an administrator, a navigator or social worker, and a fourth champion for health equity–focused systems change. The large “Other” category (*n* = 11) resulted from this fourth health care champion role. These learners included a radiation therapist (*n* = 1), a retired social worker and cancer survivor (*n* = 1), program directors (*n* = 4), public health analysts (*n* = 3), and a data coordinator (1). One participant did not identify their professional role beyond “team member.” Participants were predominantly female, white, and heterosexual with a mean age of 46.

For the study’s primary endpoints, see Table 2. There were statistically significant improvements to self-reported Cultural Competence Behaviors (*p* = .055) and Attitudinal Awareness about LGBT health (*p* = .008) from baseline to posttest. These two factors drove statistically significant findings for the overall CCA (*p* = .070) and LGBT-DOCSS (*p* = .008). No other statistically significant changes were found. Since responses were voluntary, some respondents chose not to answer some questions. To preserve power in the sample, missing data were deleted pairwise rather than listwise. This resulted in different sample sizes for different variables being measured.

**Table 2:** Outcomes of TEAM training

	*n*	Pretest mean (SD)	Posttest mean (SD)	*t*	df	*p* value
CCA (range 0–100)	17	71.647 (3.12)	76.941 (4.20)	− 2.072	16	*.070*
Cultural Competence Behaviors (range 0–68)	17	43.647 (10.48)	48.294 (13.33)	− .809	16	*.055*
Cultural Awareness and Sensitivity (range 0–32)	17	28.000 (3.73)	28.647 (4.80)	− 1.938	16	.430
LGBT-DOCSS (range 0–72)	20	43.6500 (15.07)	53.350 (9.60)	− 1.666	19	*.008*
Clinical Preparedness (range 0–28)	16	13.500 (6.96)	15.500 (6.71)	− 2.138	15	.117
Attitudinal Awareness (range 0–28)	20	23.200 (4.67)	24.800 (4.11)	− 1.667	19	*.046*
Basic Knowledge (range 0–16)	16	12.063 (3.87)	13.313 (2.75)	− 2.981	15	.116
ISVS (range 0–96)	16	78.375 (12.86)	82.250 (9.56)	− 1.320	15	.207

Statistical significance at *p* < .10 shown in italics

## Discussion

Cultural competency is complex to define, achieve, and measure. Generally, cultural competency includes awareness of the self and others, relational intelligence, and application of knowledge and lived experience to interactions with others. Cultural differences can be distinguished by race, ethnicity, sexual orientation, gender identity, socioeconomic status, education, nationality, political affiliation, religious affiliation, spirituality, degree of religiosity, and many other socially-constructed factors. It is challenging for all of us to self-reflect, identify cues from body language, respond to individual care preferences, and apply cultural humility when working with those from different lived experiences; however, doing so in the context of health care is critical.

Despite the small sample, this evaluation of the hybrid TEAM training yielded statistically significant improvement to learners’ self-reported cultural competence to care for patients of diverse racial, ethnic, sexual, gender, geographic, and other intersectional lived experiences. Specifically, improvements to attitudes in caring for sexual and gender minorities were reported. While participants noted improvements in Cultural Competence Behaviors from pre- to posttest, scores indicated that substantial room for improvement remains. Similarly, while participants reported nonsignificant improvements in Clinical Preparedness in caring for sexual and gender minorities, substantial room for improvements to learner confidence in this area remains. These findings support the need for ongoing diversity and inclusion training. It is important to note that Cultural Awareness and Sensitivity scores and Interprofessional Socialization and Valuation were high at baseline; thus, lack of statistically significant improvements was likely a result of higher baseline scores rather than failure of the intervention.

The effectiveness of the TEAM training among an interprofessional group is important, because building competence among diverse professionals within an organization provides trainees with allies that may optimize more culturally affirming care at multiple levels. A recent review of cultural competency trainings noted a dearth of evidence-based cultural competency training available to health care practitioners beyond psychology and some allied health professions [10]. Existing evaluations of cultural competency trainings in the extant literature reflect missed opportunities to highlight the importance of intersectionality and interprofessionalism in creating affirming care environments for diverse patients. Kendi suggests that any time we attribute characteristics to a group rather than an individual, we perform a racist or sexist or otherwise biased act [18]. Intersectionality emphasizes the importance of honoring the diversity of ways that people identify and pushes against group stereotypes. An intersectional approach to cultural competency training is a more nuanced approach to building healthcare professional capacity to self-reflect and champion affirming care environments for diverse patients.

The instruments selected to evaluate effectiveness of the TEAM training were chosen to reflect the training’s emphasis on intersectionality and interprofessionalism—specifically, cultural awareness and sensitivity toward diverse groups of people (CCA [14]) and valuation of learning in an interprofessional environment (ISVS [16]). A specific scale examining knowledge, attitudes, and preparedness in working with sexual and gender minorities was included given the novelty of including that content as part of the training (LGBT-DOCSS [15]). There are relatively few psychometrically validated instruments in the research literature, and the tools selected have shown strong psychometrics.

### Strengths and Limitations

A strength of this study was the use of validated measures. Additionally, this is one of the few studies examining the effectiveness of a cultural competency training on interprofessional oncology professionals. It is also one of the few interventions assessing changes to competency in working with sexual and gender minorities.

Limitations of this study include the small sample size and the high degree of missing data due to the voluntary nature of the evaluation. Missing data was also partially due to the irrelevance of clinical subscales to non-clinical learners. Finally, social desirability bias is always a limitation of self-reported data.

Future research should focus on the recruitment of larger, diverse sample sizes for comparison. Qualitative data investigating specific aspects of cultural competency training that are most effective are also warranted.

## Conclusions

This study suggests that cultural competency training among interprofessional oncology health care professionals can be effective. Given the growing diversity within the USA, current racial tensions, increasing globalization of culture, and the damage to society resulting from inequitable care, additional opportunities for diversity training are critically needed.

## Data Availability

Data is available upon request to the corresponding author.
